# Biodiversity Informatics: the emergence of a field

**DOI:** 10.1186/1471-2105-10-S14-S1

**Published:** 2009-11-10

**Authors:** Indra Neil Sarkar

**Affiliations:** 1 Center for Clinical and Translational Science, University of Vermont, Burlington, VT, USA; 2 Department of Microbiology and Molecular Genetics, College of Medicine, University of Vermont, VT, USA; 3 Department of Computer Science, College of Engineering and Mathematical Science, University of Vermont, VT, USA

## 

Recent years have seen great technological advances that have helped usher in a new generation of approaches to understand and share knowledge about the planet in which we live. A number of major initiatives that aim to catalyze necessary technological and biological advances synergistically have emerged globally. Of these enabling initiatives, the Encyclopedia of Life (EOL; ) and the Barcode of Life (BOL; ) are projects that have collectively help lay a framework within which we will see the next generation of taxonomic innovation and discovery. The successes of both EOL and BOL have the potential to impact multiple facets of society - from the discovery of new species, to the development of conservation strategies for endangered life, to insights into infectious disease hosts and vectors, to the discovery of life-saving medicinal plants, to the piquing of general interest about life on Earth and our role in the complex web of life. Both the EOL and BOL initiatives are possible thanks to a number of significant advancements in knowledge discovery, integration, and management techniques. Collectively termed 'biodiversity informatics,' this new suite of methodologies and tools extends contemporary computer science and informatics principles within the context of biodiversity data.

This supplement, made possible through funding from both EOL and BOL, brings forth some of the pioneering work from leading biodiversity informatics researchers. While nascent as a discipline, biodiversity informatics has proven to not only adopt, but also help significantly challenge and advance the most recent technological advances and computational approaches for managing complex data. In contrast to bioinformatics, which in primarily focused on managing relevant molecular biology data, biodiversity informatics requires frameworks and approaches that can accommodate the full range of biological information - from molecules to morphological features, to populations, to habitats - collectively developing the ultimate computational Web of knowledge about life on Earth.

This supplement starts with a piece from Chavan and Ingwersen that goes through some of the fundamental principles for disseminating biodiversity information, discussing both the hindrances and opportunities [1]. Hill *et al. *then describe how one might leverage existing technological infrastructure for enabling georeferencing of biodiversity data [2]. Demonstrating how biodiversity informatics can often benefit from the latest advances in searching strategies, Hajibabaei and Singer describe an approach for making use of Google to identify relevant information with respect to DNA sequences [3]. Next, Page discusses the nuances of biological global unique identifiers, which may be necessary to link relevant biodiversity data across various spheres of knowledge [4]. In consideration of the necessary continual curation required for disparate biodiversity knowledge, Smith *et al. *describe a Drupal-based technology called "Scratchpads" for managing and sharing biodiversity knowledge [5].

In parallel to the emerging infrastructure for managing and disseminating biodiversity information, DNA Barcode analysis methods represent a crucial entry point into the realm of biodiversity knowledge. The last four articles thus focus on some recent advances in DNA Barcoding analytic approaches. The first of these articles, from Bertolazzi *et al.*, presents a machine learning approach for classifying species according to DNA Barcode derived information [6]. Chu *et al. *then describe a 'composition vector' approach for making use of large datasets of DNA Barcodes for classification [7]. In light of molecular sequence alignment as an often rate-limiting step in many classification approaches, Kuksa and Pavlovic present an alignment-free approach for DNA Barcode data [8]. In light of the range of approaches associated with DNA Barcode based classification, Austerlitz *et al. *present an overview of common phylogenetic and statistical methods most commonly considered [9].

A unifying theme in the articles of this supplement is the diversity of issues that remain to be resolved going forward. It is my hope that this issue helps continue and inspire new dialogue across the full range of disciplines associated with this burgeoning field.

## Competing interests

The author declares that he has no competing interests.
